# Archaea: The First Domain of Diversified Life

**DOI:** 10.1155/2014/590214

**Published:** 2014-06-02

**Authors:** Gustavo Caetano-Anollés, Arshan Nasir, Kaiyue Zhou, Derek Caetano-Anollés, Jay E. Mittenthal, Feng-Jie Sun, Kyung Mo Kim

**Affiliations:** ^1^Evolutionary Bioinformatics Laboratory, Department of Crop Sciences, Institute for Genomic Biology and Illinois Informatics Institute, University of Illinois at Urbana-Champaign, Urbana, IL 61801, USA; ^2^School of Science and Technology, Georgia Gwinnett College, Lawrenceville, GA 30043, USA; ^3^Microbial Resource Center, Korea Research Institute of Bioscience and Biotechnology, Daejeon 305-806, Republic of Korea

## Abstract

The study of the origin of diversified life has been plagued by technical and conceptual difficulties, controversy, and apriorism. It is now popularly accepted that the universal tree of life is rooted in the akaryotes and that Archaea and Eukarya are sister groups to each other. However, evolutionary studies have overwhelmingly focused on nucleic acid and protein sequences, which partially fulfill only two of the three main steps of phylogenetic analysis, formulation of realistic evolutionary models, and optimization of tree reconstruction. In the absence of character polarization, that is, the ability to identify ancestral and derived character states, any statement about the rooting of the tree of life should be considered suspect. Here we show that macromolecular structure and a new phylogenetic framework of analysis that focuses on the parts of biological systems instead of the whole provide both deep and reliable phylogenetic signal and enable us to put forth hypotheses of origin. We review over a decade of phylogenomic studies, which mine information in a genomic census of millions of encoded proteins and RNAs. We show how the use of process models of molecular accumulation that comply with Weston's generality criterion supports a consistent phylogenomic scenario in which the origin of diversified life can be traced back to the early history of Archaea.

## 1. Introduction

“*Imagine a child playing in a woodland stream, poking a stick into an eddy in the flowing current, thereby disrupting it. But the eddy quickly reforms. The child disperses it again. Again it reforms, and the fascinating game goes on. There you have it! Organisms are resilient patterns in a turbulent flow—patterns in an energy flow*”— Carl Woese [1].

Understanding the origin of diversified life is a challenging proposition. It involves the use of ideographic thinking that is historical and retrodictive, as opposed to nomothetic explorations that are universal and predictive [2]. Experimental science for the most part is nomothetic; the search for truth comes from universal statements that can be conceptualized as being of general predictive utility. Nomothetic explorations are in general both philosophically and operationally less complex to pursue than any ideographic exploration. In contrast, retrodictions speak about singular or plural events in history that must be formalized by “transformations” that comply with a number of evolutionary axioms [3] and interface with a framework of maximization of explanatory power [4]. The fundamental statement that organismal diversity is the product of evolution is supported by an ensemble of three nested primary axioms of the highest level of universality [3]: (i) evolution occurs, including its principle that history of change entails spatiotemporal continuity (*sensu* Leibnitz), (ii) only one historical account of all living or extinct entities of life and their component parts exists as a consequence of descent with modification, and (iii) features of those entities (characters) are preserved through generations via genealogical descent. History must comply with the “principle of continuity,” which crucially supports evolutionary thinking. The axiomatic rationale of “*natura non facit saltum*” highlighted by Leibnitz, Linnaeus, and Newton must be considered a generality of how natural things change and a “fruitful principle of discovery.” We note that this axiomatic generality, which we have discussed in the context of origin of life research [5], encompasses rare punctuations (e.g., quantum leap changes such as genome duplications and rearrangements or the rare evolutionary appearance of new fold structures) embedded in a fabric of gradual change (e.g., changes induced by point mutations). Both gradual changes and punctuations are interlinked and are always expressed within spatiotemporal continuity (e.g., structural punctuations in the mappings of sequences into structures of RNA [6]). This interpretative framework can explain novelty and complexity with principles of scientific inquiry that maximize the explanatory power of assertions about retrodictions.

Phylogenetic theories are embodied in evolutionary “trees” and “models.” Trees (phylogenies) are multidimensional statements of relationship of the entities that are studied (phylogenetic taxa). Models are evolutionary transformations of the biological attributes examined in data (phylogenetic characters), which define the relationships of taxa in trees. The tripartite interaction between characters, models, and trees must occur in ways that enhance retrodictive power through test and corroboration [4]. In other words, it must follow the Popperian pillars of scientific inquiry or suitable philosophical analogs. We note that retrodictive statements allow drawing inferences about the past by using information that is extant (i.e., that we can access today) and is necessarily modern. The challenge of travelling back in time rests on not only making inferences about archaic biology with information drawn from modern biological systems but also interpreting the retrodictive statements without conceptual restrictions imposed by modernity. This has been an important obstacle to historical understanding, starting with grading hypotheses inspired by Aristotle's great chain of being, the* scala naturae*.

It was Willi Hennig in the fifties who first formalized retrodiction in quantitative terms [7]. Since then, his “phylogenetic systematics” has benefitted from numerous conceptual and bioinformatics developments, which are now responsible for modern phylogenetic analysis of systems of any kind: from molecules and organisms to language and culture, from engineering applications to astrophysics. Astrocladistics, for example, focuses on the evolution and diversification of galaxies caused by transforming events such as accretion, interaction, and mergers (e.g., [8]). While major views have emerged in the “discovery operations” (*sensu* [2]) of the phylogenetic systematics paradigm, including maximum parsimony and the frequentist and uncertainty views of maximum likelihood and Bayesian thinking, the major technical and philosophical challenges persist [9]. More importantly, as we will explain below, technical and philosophical aspects of the ideographic framework in some cases have been turned into landscapes of authoritarianism and apriorism [3]. This insidious trend is pervasive in the “rooting of the tree of life" field of inquiry [10] that underlies the origins of biochemistry and biodiversity we here discuss.

In this opinion paper we address the challenges of finding an origin to biodiversity and propose a new framework for deep phylogenetic analysis that focuses on the parts of biological systems instead of the whole. We review the application of this framework to data drawn from structural and functional genomics and argue that the origin of cellular life involved gradual accretion of molecular interactions and the rise of hierarchical and modular structure. We discuss our findings, which provide strong support to the very early rise of primordial archaeal lineages and the emergence of Archaea as the first domain of diversified cellular life (superkingdom). The term “domain” of life stresses the cohesiveness of the organism supergroup, very much like domains in proteins and nucleic acids stress the molecular cohesiveness of their atomic makeup. Instead, the term “superkingdom” (superregnum) makes explicit the fact that there is a nested hierarchy of groups of organisms, many of which share common ancestors (i.e., they are monophyletic). We propose that the rise of emerging lineages was embedded in a primordial “evolutionary grade” (*sensu* Huxley [11]), a group of diversifying organisms (primordial archaeons) in active transition that were initially unified by the same and archaic level of physiological complexity. Our discussion will attempt to reconcile some divergent views of the origin of diversified life and will provide a generic scenario for “turning points” of origin that may be recurrent in biology.

## 2. A Tripartite World of Organismal Diversity

**Figure 1:**
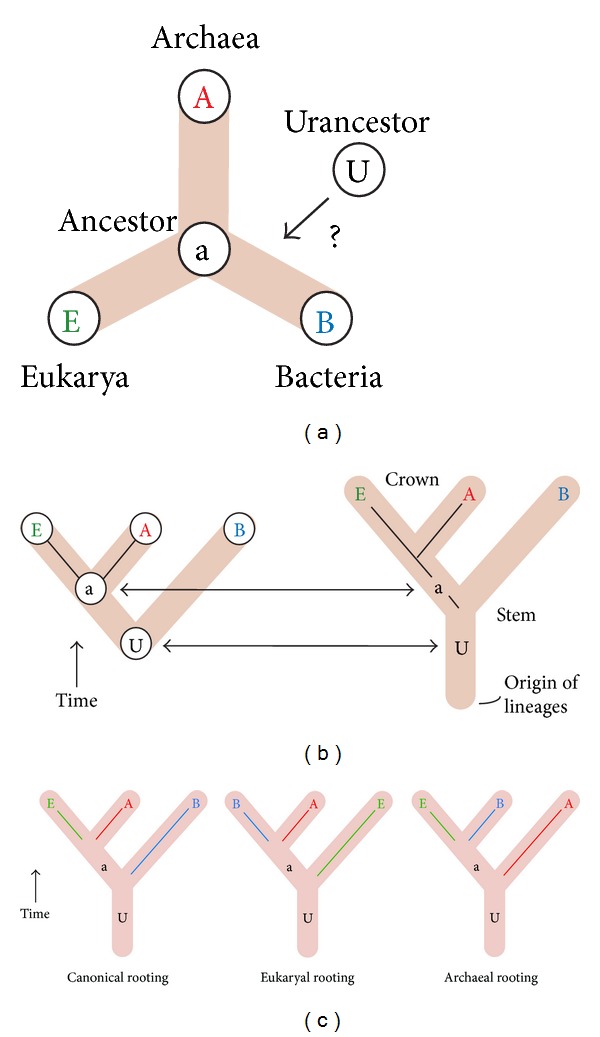
Rooting the tree of life (ToL): an exercise for “tree-thinkers.” (a) A node-based tree representation of the ToL focuses on taxa (sampled or inferred vertices illustrated with open circles) and models edges as ancestry relationships. The unrooted tree describes taxa as conglomerates of extant species for each domain of life, Archaea (A), Bacteria (B), and Eukarya (E) (abbreviations are used throughout the paper). Adding a universal ancestor vertex (rooting the ToL) implies adding a reconstructed entity (urancestor) that roots the tree but does not model ancestry relationships. The vertex is either a “most recent universal ancestor” if one defines it from an ingroup perspective or a “last universal common ancestor” (LUCA) if the definition relates to an outgroup perspective. Rooting the tree enables defining “total lineages,” which are lists of ancestors spanning from the ancestral taxon to the domain taxa. (b) A stem-based view focuses on edges (branches), which are sampled, and inferred ancestral taxa are viewed as lineages under the paradigm of descent with modification. Vertices correspond to speciation events. Terminal edges represent conglomerates of lineages leading to domains of life (the terminal nodes of node-based trees) and the ancestral stem represents the lineage of the urancestor (U) (double arrowhead line). Total lineages are simply a chain of edges that goes back in time and ends in the ancestral stem. Both node-based and stem-based tree representations are mathematically isomorphic but they are not equal [154]. They change the concept of monophyletic and paraphyletic relationships. A node-based clade starts with a lineage at the instant of the splitting event, incorporating the ancestor into the makeup of the clade. In contrast, a stem-based clade originates with a planted branch on the tree, where the branch represents a lineage between two lineage splitting events. Planting an ancestral stem defines an origin of lineages and the first speciation event in the record of life. This delimits a crown clade of two domain lineages in the stem-based tree (labeled with black lines) that includes the ancestor of the sister groups, a, and a stem domain group at its base. (c) The three possible rootings of the ToL depicted with stem-based tree representations. Terminal edges are labeled with thin lines and these conglomerate lineages can include stem and crown groups.

Carl Woese and his colleagues of the Urbana School were responsible for the groundbreaking discovery that the world of organisms was tripartite; that is, it encompassed not two but three major “domains” of cellular life (Archaea, Bacteria, and Eukarya). Two of the three “aboriginal” lines of decent were initially conceptualized as “urkingdoms” of deep origin that were microbial and qualitatively different from eukaryotic organisms [12]. They corresponded to Archaea and Bacteria. The discovery of Archaea challenged the established akaryote/eukaryote divide (we use the term “akaryote” to describe a cell without a bona fide nucleus. This term complements the word “eukaryote” (“eu,” good, and “karyon,” kernel), which is ahistorical. The new term takes away the time component of the widely used “prokaryote” (“pro” before) definition, which may be incorrect for many organisms of the microbial domains) that supported “ladder” scenarios of gradual evolution from simplistic microbes to “higher” organisms, which were tenaciously defended by molecular biologists and microbiologists of the time. Woese and Fox [12] made it clear:* “Evolution seems to progress in a “quantized” fashion. One level or domain of organization gives rise ultimately to a higher (more complex) one. What “prokaryote” and “eukaryote” actually represent are two such domains. Thus, although it is useful to define phylogenetic patterns within each domain, it is not meaningful to construct phylogenetic classifications between domains: Prokaryotic kingdoms are not comparable to eukaryotic ones.”* The discovery was revolutionary, especially because* scala naturae* deeply seated the roots of the akaryote/eukaryote divide and microbes were considered primitive forms that did not warrant equal standing when compared to the complex organization of Eukarya (see [13] for a historical account). The significance of the tripartite world was quickly realized and vividly resisted by the establishment. Its resistance is still embodied today in new proposals of origins, such as the archaeon-bacterium fusion hypothesis used to explain the rise of Eukarya (see below). It is noteworthy that the root of the universal tree of cellular organisms, the “tree of life” (ToL), was initially not the driving issue. This changed when the sequences of proteins that had diverged by gene duplication prior to a putative universal common ancestor were analyzed with phylogenetic methods and the comparisons used to root the ToL [14, 15]. Paralogous gene couples included elongation factors (e.g., EF-Tu and EFG), ATPases (*α* and *β* subunits), signal recognition particle proteins, and carbamoyl phosphate synthetases, all believed to be very ancient (reviewed in [16]). In many cases, bacterial sequences were the first to branch (appeared at the base) in the reconstructed trees, forcing archaeal and eukaryal sequences to be sister groups to each other. This “canonical” rooting scheme of the ToL (Figure 1(a)) was accepted as fact and was quickly endorsed by the supporters of the Urbana School [17]. In fact, the acceptance of the “canonical” rooting in Bacteria became so deep that it has now prompted the search for the origins of Eukarya in the molecular and physiological constitutions of the putative archaeal sister group [18]. For example, Embley and coworkers generated sequence-based phylogenies using conserved proteins and advanced algorithms to show that Eukarya emerged from within Archaea [19–21] (refer to [22] for critical analysis). Importantly, these analyses suffer from technical and logical problems that are inherent in sequence-based tree reconstructions. For example, proteins such as elongation factors, tRNA synthetases, and other universal proteins used in their analyses are prone to high substitution rates [23]. Mathematically, it leads to loss of information regarding the root of the ToL as shown by Sober and Steel [24] (refer to [25] for more discussion). On the other hand, paralogous rootings sometimes contradicted each other and were soon and rightly considered weak and unreliable [23, 26, 27]. The validity of paralogy-based rooting methodology has proven to be severely compromised by a number of problems and artifacts of sequence analysis (e.g., long branch attraction, mutational saturation, taxon sampling, horizontal gene transfer (HGT), hidden paralogy, and historical segmental gene heterogeneity). Consequently, there is no proper outgroup that can be used to root a ToL that is built from molecular sequences, and currently, there are no proper models of sequence evolution that can provide a reliable “evolutionary arrow.” Because of this fact, archaeal and eukaryal rootings should be considered equally probable to the canonical bacterial rooting (Figure 1(c)). This is an important realization that needs to be explicitly highlighted, especially because it affects evolutionary interpretations and the likelihood of scenarios of origins of diversified life.

## 3. Mining Ancient Phylogenetic Signal in Universal Molecules

**Figure 2:**
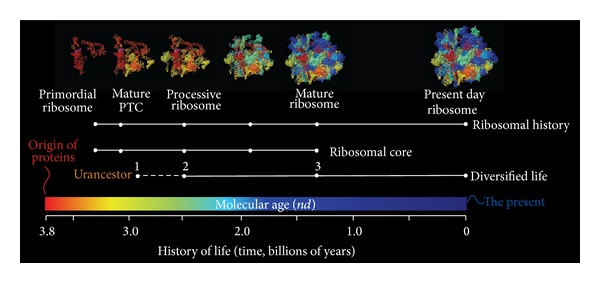
The evolutionary history of ribosome was traced onto the three-dimensional structure of its rRNA and r-protein components. Ages of components were colored with hues from red (ancient) to blue (recent). Phylogenomic history shows that primordial metabolic enzymes preceded RNA-protein interactions and the ribosome. The timeline of life derived from a universal phylogenetic tree of protein domain structure at fold superfamily level of complexity is shown with time flowing from left to right and expressed in billions of years according to a molecular clock of fold structures. The ribosomal timeline highlights major historical events of the molecular ensemble. History of the conserved ribosomal core [29] overlaps with that of diversified life [48], suggesting that episodes of cooption and lateral transfer have pervaded early ribosomal evolution.

Woese's crucial insight was the explicit selection of the ribosome for evolutionary studies. The universality of the ribosomal ensemble and its central role in protein synthesis ensured it carried an ancient and overriding memory of the cellular systems that were studied. This was made evident in the first ToL reconstructions. In contrast, many of the proteins encoded by paralogous gene couples (e.g., translation factors) likely carried convoluted histories of protein domain cooption or important phylogenetic biases induced, for example, by mutational saturation in their protein sequences. The ribosome is indeed the central feature of cellular life: the signature of “ribocells.” However, its embedded phylogenetic signatures are also convoluted. The constitution of the ribosome is heterogeneous. The ribosome represents an ensemble of 3-4 ribosomal RNA (rRNA) and ~70 protein (r-protein) molecules, depending on the species considered, and embodies multiple interactions with the cellular milieu needed for function (e.g., assembly and disassembly; interactions with the membrane of the endoplasmic reticulum). A group of 34 r-proteins is present across cellular life [28]. Ribosomal history has been shown to involve complex patterns of protein-RNA coevolution within the evolutionarily conserved core [29]. These patterns are expressed distinctly in its major constituents. While both of its major subunits evolved in parallel, a primordial core that embodied both processive and catalytic functions was established quite early in evolution. This primordial core was later accessorized with structural elements (e.g., accretion of numerous rRNA helical segments and stabilizing A-minor interactions) and r-proteins (e.g., the L7/L12 protein complex that stimulates the GTPase activity of EFG) that enhanced its functional properties. This included expansion elements in the structure of rRNA that were specific not only to subunits but also to individual domains of life.  Figure 2, for example, shows a phylogenomic model of ribosomal molecular accretion derived from the survey of protein domain structures in genomes and substructures in rRNA molecules. The accretion process of component parts of the universal core appears to have been a painstakingly slow process that unfolded during a period of ~2 billion years and overlapped with the first episodes of organismal diversification [30]. Despite of this complexity, the focus of biodiversity studies was for decades the small subunit rRNA molecule [31]. This focus has not changed much in recent years. Consequently, the history of organisms and populations is currently recounted by the information seated in the small subunit rRNA molecule. In other words, the historical narrative generally comes from only ~1% of ribosomal molecular constitution. This important and unacknowledged bias was already made explicit in early phylogenetic studies. For example, de Rijk et al. [32] demonstrated that phylogenies reconstructed from the small and large subunit rRNA molecules were different and that the reconstructions from the large subunit were more robust and better suited to establish wide-range relationships. The structure of the small and large subunits was also shown to carry distinct phylogenetic signatures [33]. However, only few evolutionary studies have combined small and large subunit rRNA for history reconstruction. Remarkably, in all of these cases phylogenetic signal was significantly improved (e.g., [34]).

## 4. Building a Tower of Babel from a Comparative Genomic Patchwork of Sequence Homologies

Molecular evolutionists were cognizant of the limitation of looking at the history of only few component parts, which by definition could be divergent. When genomic sequences became widely available, pioneers jumped onto the bandwagon of evolutionary genomics and the possibility of gaining systemic knowledge from entire repertoires of genes and molecules (e.g., [35–39]). The genomic revolution, for example, quickly materialized in gene content trees that reconstructed the evolution of genomes directly from their evolutionary units, the genes (e.g., [37, 39, 40]), or the domain constituents of the translated proteins [35, 41]. The sequences of multiple genes were also combined or concatenated in attempts to extract deep phylogenetic signal [42–44]. The results that were obtained consistently supported the tripartite world, backing-up the claims of the Urbana School. However, clues about ancestors and lineages leading to extant taxa were still missing.

With few exceptions that focused on the structure of RNA and protein molecules (see below), analyses based on genomic sequences, gene content, gene order, and other genomic characteristics were unable to produce rooted trees without the help of outgroups; additional* ad hoc* hypotheses that are external to the group of organisms being studied and generally carry strong assumptions. An “arrow of time” was not included in the models of genomic evolution that were used. As with sequence, ToLs that were generated were unrooted (Figure 1(a)) and generally rooted* a posteriori* either claiming that the canonical root was correct or making assumptions about character change that may be strictly incorrect. In a recent example, distance-based approaches were used to build universal network trees from gene families defined by reciprocal best BLAST hits [45]. These networks showed a midpoint rooting of the ToL between Bacteria and Archaea. However, this rooting involves a complex optimization of path lengths in the split networks and critically assumes that lineages evolved at roughly similar rates. This diminishes the confidence of the midpoint rooting, especially when considering the uncertainties of distances inferred from BLAST analyses and the fact that domains in genes hold different histories and rates of change. Another promising but ill-conceptualized case is the rooting of distance-based trees inferred by studying the frequency of* l*-mer sets of amino acids in proteins at the proteome level [46]. The compositional data generated a ToL that was rooted in Eukarya when randomized proteome sequences were used as outgroups. The assumption of randomness associated with the root of a ToL is however unsupported or probably wrong, especially because protein sequence space and its mappings to structure are far from random [47]. More importantly, a large fraction of modern proteins had already evolved protein fold structures when the first diversified lineages arose from the universal common ancestor of cellular life [48]. These evolved repertoires cannot be claimed to be random.

The inability of genomics to provide clear answers to rooting questions promoted (to some extent) parsimony thinking and the exploration of evolutionary differences of significance that could act as “anchors” and could impart “polarity” to tree statements [49]. As we will describe below, we applied parsimony thinking to the evolution of protein and RNA structures [41, 50], and for over a decade we have been generating rooted ToLs that portray the evolution of proteomes with increasing explanatory power. This work however has been for the most part unacknowledged. Instead, molecular evolutionists focused almost exclusively on molecular sequences in their search for solutions to the ToL problem. For example, analysis of genomic insertions and deletions (indels) that are rare in paralogous gene sets rooted the ToL between the group of Actinobacteria and Gram-negative bacteria and the group of Firmicutes and Archaea [51]. Unfortunately, little is known about the dynamics of indel generation, its biological role in gene duplication, and its influence on the structure of paralogous protein pairs (e.g., [52]). If the dynamics are close to that of sequences, indels should be considered subject to all the same limitations of sequence analyses, including long branch attraction artifacts, character independence, and inapplicable characters. Given a tree topology, parsimony was also used to optimize the evolutionary transformation of genomic features. For example, when considering the occurrence of protein domains that are abundant, variants of domain structures accumulate gradually in genomes; operationally the domain structure per se cannot be lost once it had been gained. This enabled the use of a variant of the unrooted Dollo algorithmic method (e.g., [53]); the Dollo parsimony model [54] is based on the assumption that when a biological feature that is very complex is lost in evolution it cannot be regained through vertical descent. The method however could not be applied to akaryotic genomes, as these are subject to extensive lateral gene transfer, and until recently [55] was not extended to ToL reconstructions.

Parsimony thinking was also invoked in “transition analysis,” a method that attempts to establish polarity of character change by, for example, examining homologies of proteins in proteolytic complexes such as the proteasome, membrane and cell envelope biochemical makeup, and body structures of flagella, sometimes aided by BLAST queries [56, 57]. The elaboration however is restricted to the few molecular and cellular structures that are analyzed, out of thousands that populate the akaryotic and eukaryotic cells. The approach is local, has not yet made use of an objective analytic phylogenetic framework, and does not weigh gains, losses, and transfers with algorithmic implementations. Thus, many statements can fall pray of incorrect optimizations or processes of convergence of structure and function, including cooptions that are common in metabolic enzymes [58]. Transitions also fail to consider the molecular makeup of the complexes examined (e.g., evolution of the photosynthetic reaction centers), which often hold domains with heterogeneous histories in the different organismal groups. For example, the *F*_1_/*F*_*o*_ ATP synthetase complex that powers cellular processes has a long history of accretion of domains that span almost 3.8 billion years of history, which is even older than that of the ribosome [59]. Finally, establishing the validity of evolutionary transitions in polarization schemes can be highly problematic; each transition that is studied requires well-grounded assumptions [60], some of which have not been yet properly elaborated.

**Figure 3:**
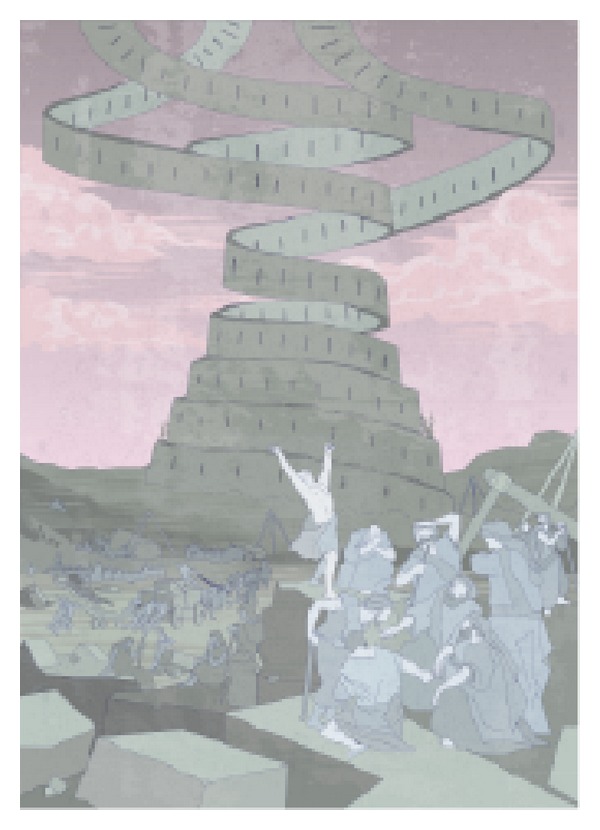
The “Confusion of Tongues,” an engraving by Gustave Doré (1832–1883), was modified by D. Caetano-Anollés to portray the event of diversification that halted the construction of the Tower of Babel. In linguistics, the biblical story inspired tree thinking. We take the metaphor as the fall of the urancestor of cellular life and the replacement of “*scala naturae*” by branching processes of complexity.

The inability to solve the rooting problem and the insistence on extracting deep phylogenetic signal from molecular sequences that are prone to mutational saturation raised skepticism about the possibility of ever finding the root of the ToL. Bapteste and Brochier [60] made explicit the conceptual difficulties claiming scientists in the field had adopted Agrippa's logic of doubt. Misunderstandings on how to conduct ideographic inquiry in evolutionary genomics had effectively blocked the reerection of a Tower of Babel that would explain the diversification of genomes (Figure 3). Instead, there was “Confusion of Doubts.” Unfortunately, the impasse was aggravated by aprioristic tendencies inherited from systematic biology [3, 10], disagreements about the evolutionary role of vertical and lateral inheritance and the problem of homology [61], and currently disagreements about the actual role of Darwinian evolution in speciation and the ToL problem [13, 62].

During the decade of evolutionary genomic discovery, the effects of HGT on phylogeny [63] took front cover. HGT was invoked as an overriding process. However, little attention was paid to alternative explanations such as differential loss of gene variants, ancient or derived, and there was little concern for other sources of reticulation. HGT is certainly an important evolutionary process that complicates the “tree” concept of phylogenetic analysis and must be carefully studied (e.g., [64–66]). In some bacterial taxonomic groups, such as the proteobacteria, HGT was found to be pervasive and challenged the definition of species [67]. Cases like these prompted the radical suggestion that the ToL should be abandoned and that a “web of life” should be used to describe the diversification of microbes and multicellular organisms [68]. However, the problem has two aspects that must be separately considered.

*(i) Mechanics.* The widespread and impactful nature of HGT must be addressed. Does its existence truly compromise the validity of phylogenetic tree statements? While HGT seems important for some bacterial lineages [69], its evolutionary impacts in Archaea and Eukarya are not as extensive (e.g., [61, 70]) and its global role can be contested [71]. In turn, the role of viruses and RNA agents in genetic exchange continues to be understudied (e.g., [72]) and could be a crucial source of reticulation. Furthermore, the relationship between differential gene loss and HGT has not been adequately formalized, especially for genes that are very ancient. Establishing patterns of loss requires establishing polarity of change and rooted trees, which as we have discussed earlier remains unattained for ToLs reconstructed from molecular sequences. 

*(ii) Interpretation*. HGT generally materializes as a mismatch between histories of genes in genomes and histories of organisms. Since genomes are by functional definition collections of genes, reticulations affect history of the genes and not of the organisms, which given evolutionary assumptions result from hierarchical relationships (e.g., [73]). Thus and at first glance, reticulation processes (HGT, gene recombination, gene duplications, and gene loss) do not obliterate vertical phylogenetic signal. The problem however remains complex. A ToL can be considered an ensemble of lineages nested within each other [74]. Protein domain lineages will be nested in gene lineages; gene lineages will be nested in lineages of gene families; gene families will be nested in organismal lineages; organisms will be nested in lineages of higher organismal groupings (populations, communities, ecosystems, and biomes), and so on. All lineage levels are defined by biological complexity and the hierarchical organization of life and follow a fractal pattern distribution, each level contributing vertical and lateral phylogenetic signal to the whole. However, sublineages in one hierarchical level may hold different histories compared to the histories of higher and lower levels. Historical mismatches introduce, for example, lineage sorting and reticulation problems that represent a complication to phylogenetic analysis. For example, mismatches between gene and organismal phylogenies exist in the presence of differential sorting of genomic components due to, for example, species extinctions or genomic rearrangements. These mismatches violate the fundamental cladistic assumption that history follows a branching process, but can be explained by* homoplasy* (convergent evolution), the horizontal “trace” of the homology-homoplasy yin-yang of phylogenetic signal. The problem of homoplasy cannot be solved by conceptually preferring one particular level of the hierarchy [74]. A focus on the organismal level, for example, will not solve the problems of hybridization that are brought by strict or relaxed sexual reproduction or viral-mediated genetic exchanges, or the problems of ancient events of fusions or endosymbiosis. However, homoplasy and the optimization of characters and trees of cladistic analysis provide a rigorous framework to discover the magnitude and source of nonvertical processes in evolution. Thus, cladistics or the ToL are not invalidated by network-like signals. In fact, recent discrete mathematical formulations that test the fundamental axioms of tree construction have also proven that the bifurcating history of trees is preserved despite evolutionary reticulations [75]. While there is no “confusion of doubts” at this level, the babel of confusion has not stopped.

Genomics revealed evolutionary patchiness, which incited hypotheses of chimeras and fusions. Phylogenies of genes were found highly discordant. Organisms that were being sequenced shared very few gene sequences, and more troublingly, gene trees that were generated possessed different topologies, especially within akaryotic organisms. With time, the number of nearly universal genes decreased and the patchiness and discordancy increased. The comparative genomic patchwork of sequence homologies showed that there were groups of genes that were only shared by certain domains of life. Thus, the makeup of genomes appeared chimeric. A number of hypotheses of chimeric origins of eukaryotes were proposed based on the fact that eukaryotes shared genes expressing sister group phylogenetic relationships with both Bacteria and Archaea [64, 76]. One that is notable is the rise of eukaryotes from a “ring of life” fusion of an archaeon and a bacterium (e.g., [77]), which in a single blow defeated both the tree-like and the tripartite nature of life. Under this school, homology searches with BLAST against a database of ~3.8 million akaryotic sequences allowed to assign archaeal, bacterial, or ambiguous ancestries to genes in the human genome and explain homology patterns as relics of the akaryotic ancestors of humans [78]. Remarkably, archaeal genes tended to be involved in informational processes, encoded shorter and more central proteins, and were less likely to be involved in heritable human diseases. The chimeric origin of eukaryotes by fusions has been rightfully contested; there is no proper evidence supporting its existence [79]. Unfortunately, chimerism opened a flood of speculations about reticulation. The history of life was seen by many through the lens of a “forest” of gene histories [80]. Reticulations and ultimately HGT were forced to explain chimeric patterns. Fusion hypotheses diverted the central issue of the rooting of the ToL and prompted the separate analysis of eukaryotic origins and akaryotic evolution.

Dagan and Martin [64] denounced that the ToL was a “tree of one percent” because only a small fraction of sequences could be considered universal and could be mined for deep phylogenetic signal. The rest would account for lateral processes that confounded vertical descent. The claim came fundamentally from networks constructed using BLAST heuristic searches for short and strong matches in genomic sequences. Phylogenetic “forests” of akaryotic genes later on boosted the claim [81]. These networks were built from 6,901 trees of genes using maximum likelihood methods. Only 102 of these trees were derived from nearly universal genes and contributed very little vertical phylogenetic signal. The initial conclusion was striking: “the original tree of life concept is obsolete; it would not even be a tree of one percent” [81]. However, the fact that vertical signal was present in the forests merited reevaluation: “replacement of the ToL with a network graph would change our entire perception of the process of evolution and invalidate all evolutionary reconstruction” [82]. We note that in these studies an ultrametric tree of akaryotes was recovered from vertical signal in a supertree of nearly universal genes. The rooted tree, which was used to simulate clock-like behavior, revealed the early divergence of* Nanoarchaeum equitans* and then Archaea [81]. While inconsistency of the forest supertree increased at high phylogenetic depths, its associated supernetwork showed there were no reticulations between Archaea and Bacteria. Thus, the deep rooting signal of archaeal diversification may be bona fide and worthy of further study.

While those that value tree thinking have contested on many grounds the idea that the ToL is “obsolete” (e.g., [13]), our take in the debate is simple. Homologies between gene sequences established through the “emperor's BLAST” (*sensu* [71]) are poor substitutes to phylogenetic tree reconstructions from gene sequences. By the same token, phylogenies reconstructed from sequences are poor substitutes to phylogenies that consider the molecular structure and function of the encoded products. Gene sequences are not only prone to mutational saturation but they generally come in pieces. These pieces represent evolutionary and structural modules that host the functions of the encoded molecules. Protein domains, for example, are three-dimensional (3D) arrangements of elements of secondary structure that fold autonomously [83], are compact [84], and are evolutionarily conserved [85, 86]. The landscape of evolutionary exploration changes when the role of protein domains in function and evolution is considered [87]. Changes at sequence level, including substitutions, insertions, and deletions, can have little impact on structure, and vice versa; sequence changes in crucial sites can have devastating consequences on function and fitness. However, there is no detailed analysis of historical mismatches between gene sequences and domain structures at the ToL level. Remarkably, while HGT seems rampant at sequence level, its impact at the domain structural level is limited [88]. This makes trees derived from domains effectively “trees of 99 percent” and their use very powerful. The fact that domains diversify mostly by vertical descent (e.g., [89–91]) suggests that gene reticulations simply reflect the pervasive and impactful combinatorial effect of domain rearrangements in proteins [92] and perhaps little else. This important claim must be carefully evaluated. It offers the possibility of dissecting how levels of organization impact processes of inheritance in biology.

## 5. Out of the Impasse: Parts and Wholes in the Evolving Structure of Systems

The rooting of the ToL is clearly muddled by the high dynamic nature of change in protein and nucleic acid sequences and by the patchiness and reticulation complexities that exist at gene level. However, it is also possible that the problems of the ToL are ultimately technical. We have made the case that the use of molecular sequences is problematic on many grounds, including mutational saturation, definition of homology of sites in sequence alignments, inapplicable characters, taxon sampling and tree imbalance, and different historical signatures in domains of multidomain proteins [93]. In particular, violation of character independence by the mere existence of atomic structure represents a very serious problem that plagues phylogenetic analysis of sequences. We here present a solution to the impasse. We show that the ToL can be rooted with different approaches that focus on structure and function and that its root is congruently placed in Archaea.

Epistemologically, phylogenetic characters must comply with symmetry breaking and the irreversibility of time [94]. In other words, characters must establish transformational homology relationships and serve as independent evidential statements. 

*(i) Characters Must Be Homologous and Heritable across Tree Terminal Units (Taxa).* Character homology is a central and controversial concept that embodies the existence of historical continuity of information [74]. Characters are “basic” evidential statements that make up columns in data matrices used for tree reconstruction. They are conjectures of perceived similarities that are accepted as fact for the duration of the study, are strengthened by Hennigian reciprocal illumination, and can be put to the test through congruence with other characters, as these are fit to the trees. To be useful, characters must be heritable and informative across the taxa rows of the data matrix. This can be evaluated, for example, with the cladistics information content (CIC) measure [95]. Finding informative characters can be particularly challenging when the features that are studied change at fast pace and when taxa sample a wide and consequently deep phylogenetic spectrum. When building a ToL, the highly dynamic nature of change in the sequence makeup of protein or nucleic acid molecules challenges the ability to retrieve reliable phylogenetic signatures across taxa, even if molecules are universally distributed and harbor evolutionarily conserved regions with deep phylogenetic signal (e.g., rRNA). The reason is that given enough time, functionally or structurally constrained regions of the sequence will be fixed (will be structurally canalized [6]) and will offer little if any phylogenetically meaningful signal to uncover, for example, the universal rRNA core. In turn, mutational saturation of unconstrained regions will quickly erase history.

The mutational saturation problem was made mathematically explicit by Sober and Steel [24] using “mutual information” and the concept of time as an information destroying process. Mutual information *I*(*X*, *Y*) between two random variables *X* and *Y* is defined by

(1) $$\begin{array}{c} I\left(X,Y\right)=\overset{}{\underset{x,y}{\sum }}\;P\left(X=x,Y=y\right)\log\left(\frac{P\left(X=x,Y=y\right)}{P\left(X=x\right)P\left(Y=y\right)}\right). \end{array}$$

When *I*(*X*, *Y*) approaches 0, *X* and *Y* become independent and no method can predict *X* from knowledge of *Y*. Importantly, mutual information approaches 0 as the time between *X* and *Y* increases in a Markov chain. Regardless of the use of a parsimony, maximum likelihood, or Bayesian-based framework of analysis, *I*(*X*, *Y*) will be particularly small when sequence sites are saturated by too many substitutions due to high substitution rates or large time scales. Ancestral states at interior nodes of the trees cannot be established with confidence from extant information even in the most optimal situation of knowing the underlying phylogeny and the model of character evolution. Under simple models, the problem is not mitigated by the fact that the number of terminal leaves of trees and the sources of initial phylogenetic information increases with time. Since a phase transition occurs when substitution probabilities exceed a critical value [96], one way out of the impasse is to find features in sufficient number that change at much slower pace than sequence sites and test if mutual information is significant and overcomes Fano's inequality. These features exist in molecular biology and have been used for phylogenetic reconstruction. They are, for example, the 3D fold structures of protein molecules [87] or the stem modules of RNA structures [97], features that change at very slow rate when compared to associated sequences. For example, protein 3D structural cores evolve linearly with amino acid substitutions per site and change at 3–10 times slower rates than sequences [98]. This high conservation highlights the evolutionary dynamics of molecular structure. Remarkably, rates of change of proteins performing a same function are maintained by functional constraints but accelerate when proteins perform different functions or contain indels. In turn, fold structural diversity explodes into modular structures at low sequence identities probably triggered by functional diversification. Within the context of structural conservation, the fact that fold structures are structural and evolutionary modules that accumulate in proteomes by gene duplication and rearrangements and spread in biological networks by recruitment (e.g., [99]) also provides a solution to the problems of vanishing phylogenetic signal. Since fold accumulation increases with time in the Markov chain, mutual information must increase, reversing the “data processing inequality” that destroys information and enabling deep evolutionary information. 

*(ii) Characters Must Show at Least Two Distinct Character States.* One of these two states (transformational homologs) must be ancestral (the “plesiomorphic” state) and the other must be derived (the “apomorphic” state) [74]. Only shared and derived features (synapomorphies) provide vertical phylogenetic evidence. Consequently, determining the relative ancestry of alternative character states defines the polarity of character transformations and roots the underlying tree. This is a fundamental property of phylogenetic inference. Polarization in tree reconstruction enables the “arrow of time” (*sensu* Eddington's entropy-induced asymmetry), solves the rooting problem, and fulfills other epistemological requirements.

Cladistically speaking, character polarity refers to the distinction between the ancestral and derived states and the identification of synapomorphies. However, an evolutionary view of polarity also refers to the direction of character state transformations in the phylogenetic model. Historically, three accepted alternatives have been available for rooting trees [100–102], the* outgroup comparison,* the* ontogenetic method,* and the* paleontological or stratigraphic method*. While the three methods do not include assumptions of evolutionary process, they have been the subject of much discussion and their interpretation of much controversy. The first two are however justified by the assumption that diversity results in a nested taxonomic hierarchy, which may or may not be induced by evolution. We will not discuss the stratigraphic method as it relies on auxiliary assumptions regarding the completeness of the fossil record. The midpoint rooting criterion mentioned earlier will not be discussed either. The procedure is contested by the existence of heterogeneities in rates of change across the trees and problems with the accurate characterization of phylogenetic distances.

In outgroup comparison, polarity is inferred by the distribution of character states in the ingroup (group of taxa of interest) and the sister group (taxa outside the group of interest). In a simple case, if the character state is only found in the ingroup, it must be considered derived. Outgroup comparison is by far the method of choice because phylogeneticists tend to have confidence in the supporting assumptions: higher-level relationships are outside the ingroup, equivalent ontogenetic stages are compared, and character state distributions are appropriately surveyed. Unfortunately, the method is “indirect” in that it depends on the assumption of the existence of a higher-level relationship between the outgroup and the ingroup. Consequently, the method cannot root the ToL because at that level there is no higher-level relationship that is presently available. Moreover, the method in itself does not polarize characters. It simply connects the ingroup to the rest of the ToL [100].

The ontogenetic criterion confers polarity through the distribution of the states of homologous characters in ontogenies of the ingroup, generally by focusing on the generality of character states, with more widely distributed states being considered ancestral. Nelson's rule states* “given an ontogenetic character transformation from a character observed to be more general to a character observed to be less general, the more general character is primitive and the less general advanced”* [103]. This “biogenetic law” appears powerful in that it depends only on the assumption that ontogenies of ingroup taxa are properly surveyed. It is also a “direct” method that relies exclusively on the ingroup. Consequently, it has the potential to root the ToL. Unfortunately, Nelson's “generality” has been interpreted in numerous ways, especially as it relates to the ontogenetic sequence, leading to much confusion [102]. It also involves comparison of developmentally nested and distinct life history stages, making it difficult to extend the method (originally conceptualized for vertebrate phylogeny) to the microbial world. However, Weston [100, 104] made it clear that the ontogenetic criterion embodies a wider “generality criterion” in which the taxic distribution of a character state is a subset of the distribution of another. In other words, character states that characterize an entire group must be considered ancestral relative to an alternative state that characterizes a subset of the group. Besides the centrality of nested patterns, the generality criterion embeds the core assumption that every homology is a synapomorphy in nature's nested taxonomic hierarchy and that homologies in the hierarchy result from additive phylogenetic change [100]. Weston's more general rule therefore states that* “given a distribution of two homologous characters in which one, x, is possessed by all of the species that possess its homolog, character y, and by at least one other species that does not, then y may be postulated to be apomorphous relative to x”* [104]. The only assumption of the method is that relevant character states in the ingroup are properly surveyed. This new rule crucially substitutes the concept of ontogenetic transformation by the more general concept of homology and additive phylogenetic change, which can be applied to cases in which homologous entities accumulate “iteratively” in evolution (e.g., generation of paralogous genes by duplication). Since horizontally acquired characters (xenologs) are not considered synapomorphies, they contribute towards phylogenetic noise and are excluded after calculation of homoplasy and retention indices (i.e., measures of goodness of fit of characters to the phylogeny).

**Figure 4:**
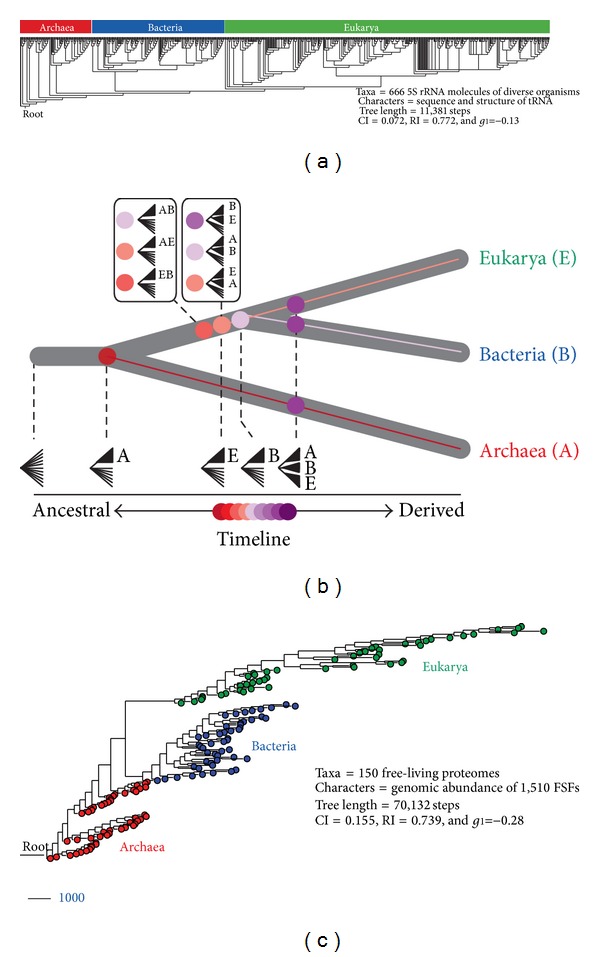
Trees of life generated from the structure of RNA and protein molecules congruently show a rooting in Archaea. (a) A rooted phylogenetic tree of 5S rRNA reconstructed from both the sequence and the structure of the molecules (from [105]). (b) Global most-parsimonious scenario of organismal diversification based on tRNA (from [107]). A total of 571 tRNA molecules with sequence, base modification, and structural information were used to build a ToL, which failed to show monophyletic groupings. Ancestries of lineages were then inferred by constraining sets of tRNAs into monophyletic groups representing competing (shown in boxes) or noncompeting phylogenetic hypotheses and measuring tree suboptimality and lineage coalescence (illustrated with color hues in circles). (c) A ToL reconstructed from the genomic abundance counts of 1,510 FSFs as phylogenetic characters in the proteomes of 150 free-living organisms sampled equally and randomly from the three domains of life (data taken from [122, 123]). Taxa were labeled with circles colored according to superkingdom. CI = consistency index, RI = retention index, and *g*_1_ = gamma distribution parameter.

We have applied the “generality criterion” to the rooting of the ToL through polarization strategies that embody axioms of evolutionary process.  Figure 4 shows three examples. A rooted phylogeny describing the evolution of 5S rRNA molecules sampled from a wide range of organisms was reconstructed from molecular sequence and structure [105]. The ToL that was recovered was rooted paraphyletically in Archaea (Figure 4(a)). The model of character state transformation was based on the axiom that evolved RNA molecules are optimized to increase molecular persistence and produce highly stable folded conformations. Molecular persistence materializes in RNA structure in vitro, with base pairs associating and disassociating at rates as high as 0.5 s^−1^ [106]. The frustrated kinetics and energetics of this folding process enable some structural conformations to quickly reach stable states. This process is evolutionarily optimized through structural canalization [6], in which evolution attains molecular functions by both increasing the average life and stability of selected conformations and decreasing their relative number. Thus, conformational diversity measured, for example, by the Shannon entropy of the base-pairing probability matrix or features of thermodynamic stability act as “evo-devo” proxy of a generality criterion for RNA molecules, in which the criterion of similarity (e.g., ontogenetic transformation) is of positional and compositional correspondence. Using a different approach, we recently broadened the use of phylogenetic constraint analysis [107, 108], borrowing from a formal cybernetic method that decomposes a reconstructable system into its components [109], and used it to root a ToL derived from tRNA sequence and structure (Figure 4(b)). The ToL was again rooted in Archaea. The number of additional steps required to force (constrain) particular taxa into a monophyletic group was used to define a lineage coalescence distance (*S*) with which to test alternative hypotheses of monophyly. These hypotheses were then ordered according to *S* value in an evolutionary timeline. Since *S* records the relative distribution of character states in taxic sets, it also embodies the generality criterion of rooting. Finally, we generated rooted ToLs that describe the evolution of proteomes directly from a census of protein fold structures in proteomes (Figure 4(c)). This ToL shows a paraphyletic rooting in Archaea. The method extracts phylogenetic signal from proteomic abundance of protein fold structures and considers that the most abundant and widely distributed folds are of ancient origin when defining transformation series [41]. This polarization scheme, which results in the gradual growth of the proteome repertoire, again represents an embodiment of the generality criterion in which statements of homology (fold structures) result from additive phylogenetic change (increases in abundance). It is noteworthy that these ToL reconstructions take into account the genomic abundance of each and every fold structure in each proteome and across the entire matrix, thereby generating a frustrated system. In general, very high abundance of only few folds will not attract taxa (species) to the derived branches in the ToL. The ancestry of taxa is determined by both the abundance and interplay among fold structure characters. For example, metabolic folds such as those involved in ATP hydrolysis are widespread in cells and considered to be very ancient. These are also the most ancient folds in our phylogenies. In comparison, some popular eukaryote-specific folds (e.g., immunoglobulin superfamilies) are highly abundant but appear in a derived manner in our phylogenies. Thus we reason that there is no circularity involved in the character polarization scheme.

The compositional schemes extend the concept of rooting with paralogous sequences to the entire proteome complements, from gene family level [100] to structural hierarchies. The three examples make use of different rooting rationales but provide a congruent scenario of origins of diversified life. Technically, roots are inferred by the Lundberg method [110] that does not require any outgroup taxa specification. This method roots the trees* a posteriori* by attaching the hypothetical ancestor to that branch of the unrooted network that would yield minimum increase in the tree length (thus preserving the principle of parsimony). 

*(iii) Characters Must Serve as Independent Evolutionary Hypotheses.* Valid phylogenetic optimization requires that characters be independent pieces of evidence. Characters should not depend on other characters. When the assumption of independence is violated, characters are overweighed in the analysis and the resulting phylogeny fails to represent true history [111]. Possible dependencies could be of many kinds, from structural to functional, from developmental to ecological. These dependencies distort and obscure phylogenetic signal and must be either avoided or coded into the phylogenetic model through parameters or weight corrections.

As we will now elaborate, the problem of character independence is about* parts* and* wholes* in the hierarchical fabric of life and in the nested hierarchies of the ToL. Biological systems are by definition made of parts regardless of the way parts are defined. In evolution, diversification and integration of parts unify parts into cohesive entities, modules, which then diversify [112]. This process and the rise of modules may explain evolutionary waves of complexity and organization and the emergence of structure (defined broadly) in biology that is hierarchical. The hierarchical makeup is made evident in the structure of protein molecules, where lower level parts of the polymer (the amino acid residues) interact with each other and establish cohesive higher-level modular parts, which also establish interaction networks and are crucial for molecular function and for interaction of proteins with the cellular environment. Consequently, the structure of proteins can be described at increasing hierarchical levels of structural abstraction: sequences, motifs, loops, domains, families, superfamilies, topologies, folds, architectures, and classes. Two accepted gold standards of protein classification, structural classification of proteins (SCOP) [113] and class architecture topology homology (CATH) [114], use parts of this incomplete scheme to describe the atomic complexity of the molecules. We note that these classifications do not consider unrealized structural states, such as protein folds that are possible but that have never been identified in the natural world of protein structures. We also note that modules sometimes engage in combinatorial games. For example, protein domains are rearranged in evolution by fusions and fissions producing the enormous diversity of alternative domain rearrangements that exist in multidomain proteins [92].

**Figure 5:**
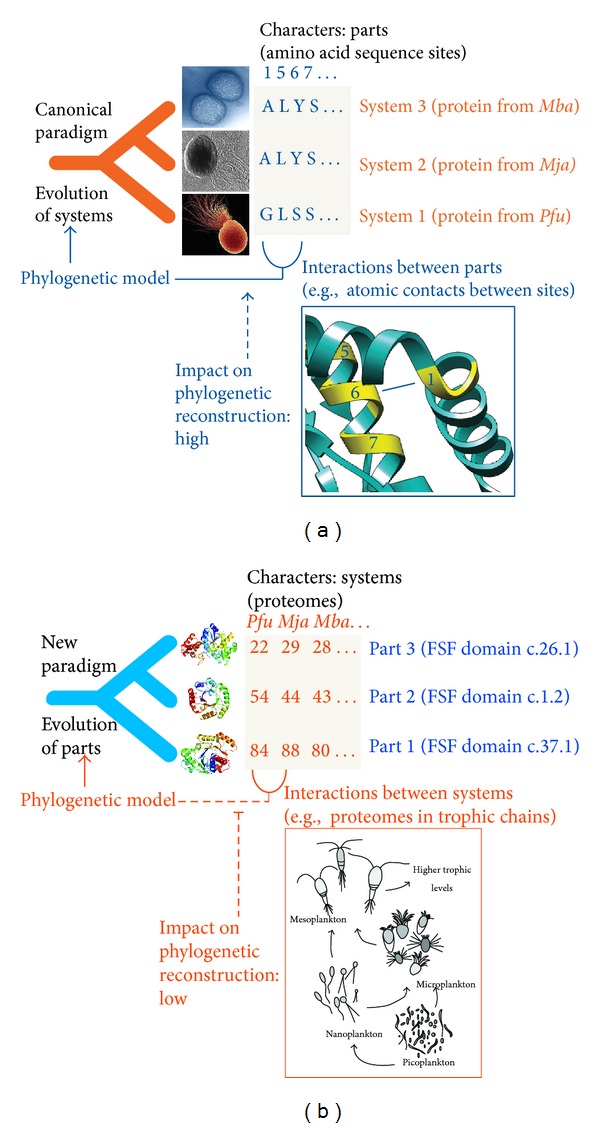
A new phylogenetic strategy simplifies the problems of character independence. (a) The canonical paradigm explores the evolution of systems, such as the evolution of organisms in the reconstruction of ToLs. For example, the terminals of phylogenetic trees can be genes sampled from different organisms (e.g.,* Pyrococcus furiosus* (*Pfu*),* Methanococcus jannaschii* (*Mja*), and* Methanosarcina barkeri* (*Mba*)), and phylogenetic characters can be amino acid sequence sites of the corresponding gene products. Character states can describe the identity of the amino acid at each site. Since characters are molecular parts that interact with other parts when molecules fold into compact 3D structures, their interaction violates the principle of character independence. Consequently, the effects of covariation must be considered in the phylogenetic model used to build the trees. (b) The new paradigm explores the evolution of parts, such as the evolution of protein domains in proteomes. For example, the terminals of phylogenetic trees can be domains defined at fold superfamily level of structural complexity and the characters used to build the trees can be proteomes. Character states can be the number of domains holding the FSF structure. Since proteomes interact with other proteomes when organisms establish close interactions, interactions that could affect the abundance of domains in proteomes should be considered negligible (unless there is an obligate parasitic lifestyle involved) and there is no need to budget trophic interactions in the phylogenetic model.

Since biological systems evolve and carry common ancestry, parts of these systems by definition evolve and by themselves carry common ancestry. In other words, the histories of parts are embodied in the ensemble of lineages of the ToL. The focus of phylogenetic analysis however has been overwhelmingly the organism as the biological system, as testified by the effort devoted to taxonomical classification, systematic biology, and building of the ToL. The genomic revolution has provided a wealth of parts in the amino acid sequence sites of proteins and nucleic acid molecules, which have been used as phylogenetic characters for analysis of molecules representing organisms. This has maintained the focus of reconstructing trees of systems (the wholes). For example, amino acid sites of a protein are generally fit into a data matrix (alignment), which is then used to build trees of genes and organisms (Figure 5(a)) using modern algorithmic implementations [115]. However and as we have mentioned, proteins have complicated structures that result from interactions between amino acids at the 3D atomic level. These intramolecular interactions, or at least some of them [116], induce protein folding and delimit molecular function and protein stability. They are responsible for protein secondary, supersecondary, domain, and tertiary structure, and, by definition, their mere existence induces violation of character independence. Penny and Collins [117] proposed the simple thought experiment in which the bioinformatician exchanges rows of sequence sites in the alignment matrix and asks what was lost in the process. Randomization of characters (columns) in the data matrix does not change the phylogenetic tree. However, randomization destroys the structure of the molecule and very likely its function. This confirms that reconstruction of trees from information in sequence sites violates character independence, and in the process ignores structure and biology. The effects of violation of character independence may be minimal for trees of sequences that are closely related. However, trees describing deep historical relationships require that sequences be divergent and this maximizes the chances of even wider divergences in molecular structure that are not being accounted for by the models of sequence evolution.

The genomic revolution has also provided a wealth of models of 3D atomic structure. These structures are used as gold standards to assign with high confidence structural modules to sequences. As mentioned earlier, proteomes embody collections of protein domains with well-defined structures and functions. Protein domain counts in proteomes have been used to generate trees of protein domains (Figure 5(b)) using standard cladistic approaches and well-established methods (reviewed in [87]). These trees describe the evolution of protein structure at global level. They are effectively trees of parts. While domains interact with each other in multidomain proteins or establish protein-protein interactions with the domains of other proteins, these interactions of parts do not violate character independence. This is because phylogenetic characters are actually proteomes, systems defined by structural states that exist at much higher levels than the protein domain, not far away from the organism level. Remarkably, no information is lost when character columns in the matrix are randomized in the thought experiment. The order of proteome characters in the matrix does not follow any rationale. Characters are not ordered by lifestyles or trophic levels of the organisms. Interactions between free-living organisms will seldom bias their domain makeup, and if so, those characters can be excluded from analysis. Even the establishment of symbiotic or obligate parasitic interactions, such as the nodule-forming symbioses between rhizobia and legumes, may have little impact on character independence, as long as the joint inclusion of the host and the symbiont is avoided.

## 6. Evidence Supporting the Archaeal Rooting of the Universal Tree

Figure 4 shows examples of rooted ToLs generated from the sequence and structure of RNA and protein molecules. The different phylogenomic approaches arrive at a common rooted topology that places the stem group of Archaea at the base of the ToL (the archaeal rooting of Figure 1(c)). However, the evolutionary interrelationship of parts and wholes prompt the use of trees of parts, a focus on higher-level structure, and a decrease in confidence in the power of trees of systems. These concepts have been applied to the study of the history of nucleic acid and protein structures for over a decade and provide additional evidence in support of the archaeal rooting scenario.

### 6.1. Comparative Genomic Argumentation

**Figure 6:**
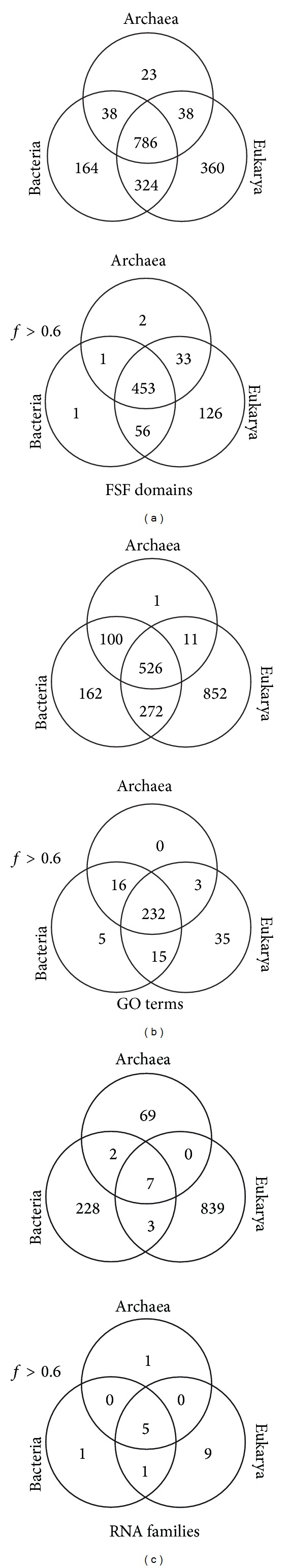
Venn diagrams displaying the distributions of 1,733 FSF domains (a), 1,924 terminal GO terms (b), and 1,148 RNA families (c) in the genomes of the three domains of life. FSF domain data was taken from Nasir et al. [122, 123] and included 981 completely sequenced proteomes from 70 Archaea, 652 Bacteria, and 259 Eukarya. Terminal GO terms corresponding to the “molecular function” hierarchy defined by the GO database [118] were identified in 249 free-living organisms, including 45 Archaea, 183 Bacteria, and 21 Eukarya (data taken from [123]). The Venn diagram of RNA families and the distribution of Rfam clans and families in organisms were taken from Hoeppner et al. [129] and their Dataset S1. Shown below are distribution patterns for FSFs, GO terms, and RNA families that are present in more than 60% of the organisms examined (*f* > 0.6). All distributions highlight maximum sharing in the ancient ABE and BE taxonomic groups and minimal sharing in archaeal taxonomic groups.

The distribution of gene-encoded products in the genomes of sequenced organisms and parsimony thinking can reveal global evolutionary patterns without formal phylogenetic reconstruction. We will show how simple numerical analyses of protein domains, molecular activities defined by the gene ontology (GO) consortium [118], and RNA families that are domain-specific or are shared between domains of life can uncover the tripartite division of cellular life, exclude chimeric scenarios of origin, and provide initial insight on the rooting of the ToL (Figure 6).

**Figure 7:**
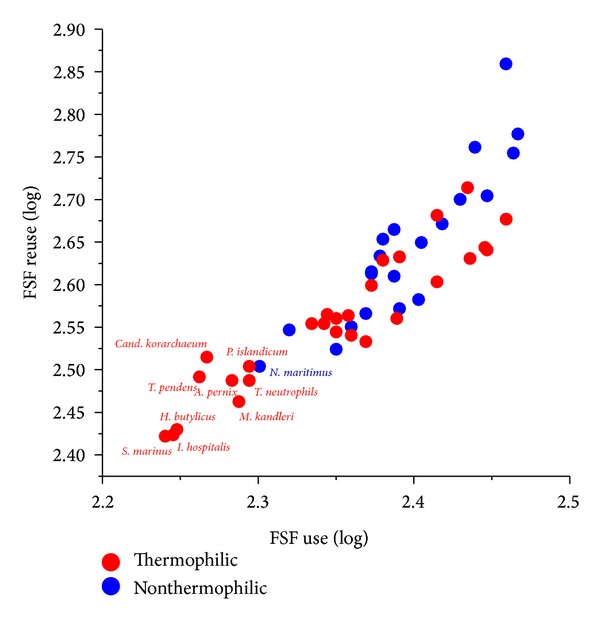
A plot of FSF use (diversity) against FSF reuse (abundance) reveals a linear pattern of proteomic growth in 48 archaeal proteomes. Thermophilic archaeal species occupy positions that are close to the origin of the plot. They also populate the most basal branch positions in ToLs (see discussion in the main text). Both axes are in logarithmic scale.

The number of unique protein folds observed in nature is very small. SCOP ver. 1.75 defines only ~2,000 fold superfamilies (FSFs), groups of homologous domains unified on the base of common structural and evolutionary relationships, for a total of 110,800 known domains in proteins [113, 119]. FSFs represent highly conserved evolutionary units that are suitable for studying organismal diversification [87]. Yafremava et al. [120] plotted the total number of distinct FSFs (FSF diversity) versus the average reuse of FSFs in the proteome of an organism (FSF abundance). This exercise uncovered a scaling behavior typical of a Benford distribution with a linear regime of proteomic growth for microbial organisms and a superlinear regime for eukaryotic organisms (Figure 8 in [120]). These same scaling patterns are observed when studying the relationship between open reading frames and genome size [121]. Remarkably, archaeal and eukaryal proteomes exhibited both minimum and maximum levels of FSF abundance and diversity, respectively. Bacterial proteomes however showed intermediate levels. We note that the general scaling behavior is consistent with a scenario in which evolutionary diversification proceeds from simpler proteomes to the more complex ones in gradual manner, supporting the principle of spatiotemporal continuity and revealing the nested phylogenetic hierarchies of organisms. Under this scenario (and results of [120]), the streamlined archaeal proteomes represent the earliest form of cellular life. Remarkably, archaeal species harboring thermophilic and hyperthermophilic lifestyles encoded the most streamlined FSF repertoires (Figure 7). Clearly, modern thermophilic archaeons are most closely related to the ancient cells that inhabited planet Earth billions of years ago (also read below).

FSF domain distributions in the genomes of the three domains of life provide further insights into their evolution. Nasir et al. [122, 123] generated Venn diagrams to illustrate FSF sharing patterns in the genomes of Archaea, Bacteria, and Eukarya (Figure 6(a)). These diagrams display the total number of FSFs that are unique to a domain of life (taxonomic groups A, B, and E), shared by only two (AB, BE, and AE), and those that are universal (ABE). About half of the FSFs (786 out of 1,733) were present in all three domains of life, 453 of which were present in at least 60% of the organisms that were examined (*f* > 0.6, where *f* is the distribution index ranging from 0 to 1). The fact that about 70% of widely shared FSFs (672) belong to the ABE taxonomic group strongly supports a common evolutionary origin for cells. Evolutionary timelines have confirmed that a large number of these universal FSFs were present in the ancestor of the three domains of life, the urancestor (Figure 1), and were for the most part retained in extant proteomes [48].

Interestingly, the number of BE FSFs was ~10-fold greater than AE and AB FSFs (324 versus 38 and 38) (Figure 6(a)). The finding that Bacteria and Eukarya encode a significantly large number of shared FSF domains is remarkable and hints towards an unprecedented strong evolutionary association between these two domains of life. Moreover, a significant number of BE FSFs is widespread in the bacterial and eukaryotic proteomes; 56 of the 324 FSFs are shared by more than 60% of the bacterial and eukaryal organisms that were analyzed (Figure 6(a)). This is strong evidence of an ancient vertical evolutionary trace from their mutual ancestor (anticipated in [123]). This trace uniquely supports the archaeal rooting of the ToL. In turn, the canonical and eukaryotic rooting alternatives are highly unlikely as none alone can explain the remarkable diversity of the BE taxonomic group (as well as its ancient origin; [123]). The remarkable vertical trace of the ABE taxonomic group and the negligible vertical trace of the AB group (only one FSF shared by more than 60% of organisms) also falsify fusion hypotheses responsible for a putative chimeric makeup of Eukarya. In light of these findings, the most parsimonious explanation of comparative genomic data is that Archaea is the first domain of diversified cellular life.

The patterns of FSF sharing are further strengthened by the genomic distributions of terminal-level molecular function GO terms (Figure 6(b)). The three domains of life shared about a quarter (526) of the 1,924 GO terms that were surveyed. A total of 232 of these ABE terms were shared by 60% of organisms analyzed. Again, the fact that about 76% of widely shared GO terms (306) belong to the ABE taxonomic group strongly supports a common vertical trace, while the finding that Bacteria and Eukarya share a significantly large number of GO terms (272) supports again the archaeal rooting of the ToL. An alternative explanation for the very large size of BE could be large-scale metabolism-related gene transfer from the ancestors of mitochondria and plastids to the ancestors of modern eukaryotes [124]. However, we note that the BE group is not restricted to only metabolic functions. It also includes FSFs and GOs involved in intracellular and extracellular processes and regulation and informational functions [125]. Thus the very large size of BE is a significant outcome most likely shaped by vertical evolutionary scenarios and cannot solely be explained by parasitic/symbiotic relationships that exist between Bacteria and Eukarya [123]. Moreover, the simplicity of archaeal FSF repertoires is not due to the paucity of available archaeal genomic data. We confirmed that the mean FSF coverage (i.e., number of proteins/annotated to FSFs/GOs out of total) for Archaea, Bacteria, and Eukarya was largely comparable (e.g., Table S1 in [122]). Finally, as we will describe below, these comparative patterns and tentative conclusions are confirmed by phylogenomic analysis [91, 122, 126]. Congruent phylogenies were obtained with (Figure 4(c)) and without equal and random sampling of taxa. Thus, the relatively low number of archaeal genomes is also not expected to compromise our inferences.

We note that Eukarya shares many informational genes with Archaea and many operational genes with Bacteria. While informational genes have been thought more refractory to HGT than noninformational genes, we have confirmed at GO level that this is not the case. A ToL built from GO terms showed that in fact noninformational terms were less homoplasious, while statistical enrichment analyses revealed that HGT had little if any functional preference for GO terms across GO hierarchical levels. We recently compared ToLs reconstructed from non-HGT GO terms and ToLs reconstructed from informational GO terms that were extracted from non-HGT GO terms (Kim et al. ms. in review; also read below). In both cases, Archaea appeared as a basal paraphyletic group of the ToLs and the common origin of Bacteria and Eukarya was maintained. Thus, the 272 GO terms shared by Bacteria and Eukarya harbor a strong vertical trace.

Another observation often used as support for Archaea-Eukarya kinship is the discovery of few eukaryote-specific proteins (e.g., actin, tubulin, H3, H4, ESCRT, ribosomal proteins, and others) in some archaeal species [127] and their complete absence from Bacteria (except for documented tubulin HGT from eukaryotes to bacterial genus* Prosthecobacter*; [128]). This suggests either that eukaryotes arose from an archaeal lineage [127] or that the ancestor of Eukarya and Archaea was complex and modern Archaea are highly reduced [25]. Indeed, few eukaryote-specific proteins have now been found in some archaeal species (in some cases just one species!). We argue that this poor spread cannot be taken as evidence for the Archaea-Eukarya sister relationship. This needs to be confirmed by robust phylogenetic analysis, which is unfortunately not possible when using protein sequences. Recently, we described a new strategy for inferring vertical and horizontal traces [123]. This method calculates the spread of FSFs and GOs that are shared between two-superkingdom groups (i.e., AB, AE, and BE). Balanced distributions often indicate vertical inheritance while biased distributions suggest horizontal flux. For example, penicillin binding molecular activity (GO: 0008658) was present in 100% of the sampled bacterial proteomes but was only present in 11% of the archaeal species [123]. Thus, presence of GO: 0008658 in Archaea was attributed to HGT gain from Bacteria. Using this simple method we established that both Bacteria and Eukarya were united by much stronger vertical trace than either was to Archaea. In fact, strong reductive tendencies in the archaeal genomes were recorded [123]. Thus, in our opinion, presence of eukaryote-specific proteins in only very few archaeal species could in fact be an HGT event that is not detectable by sequence phylogenies.

In turn, many arguments favor now the Bacteria-Eukarya sisterhood in addition to the structure-based phylogenies and balanced distribution of molecular features. For example, Bacteria and Eukarya have similar lipid membranes that can be used as argument for their evolutionary kinship. Moreover, Archaea fundamentally differ from Eukarya in terms of their virosphere. Viruses infecting Archaea and Eukarya are drastically different, as recently discussed by Forterre [25].

The genomic distribution of RNA families was taken from Hoeppner et al. [129] and also shows a vertical evolutionary trace in five crucial Rfam clans that are universal, including tRNA, 5S rRNA, subunit rRNA, and RNase P RNA. These universal RNA groups are likely minimally affected by HGT. However, 99% of Rfam clans and families are specific to domains of life and only 11 of the 1,148 groups were shared at *f* > 0.6 levels. This clearly shows that the functional complexity of RNA materialized very late during organismal diversification and that it is not a good genomic feature for exploring the rooting of the ToL. Only five RNA families are shared between two domains of life, and only one of these does so at *f* > 0.6, the G12 pseudoknot of the 23S rRNA, which is present in bacterial and eukaryotic organellar rRNA. While the large subunit rRNA scaffold supports the G12 pseudoknot with its vertical trace, all five interdomain RNA families can be explained most parsimoniously by HGT. Thus, only a handful of ancient and universal RNA species can be used to root the ToL.

We end by noting that the Venn diagrams consistently show that Archaea harbors the least number of unique (A) and shared (AB and AE) FSFs, GO terms or RNA families. This trend supports an early divergence of this domain of life from the urancestor and the possibility that such divergence be shaped by evolutionary reductive events. We reason that such losses would be more parsimonious early on in evolution than in the later periods. This is because genes often increase their abundance in evolutionary time (by gene duplications, HGT, and other processes). Thus it is reasonable to think that loss of an ancient gene would be more feasible very early in evolution relative to losing it very late.

### 6.2. Phylogenomic Evidence from the Sequence and Structure of RNA

Phylogenetic analyses of the few RNA families that are universal and display an important vertical evolutionary trace (Figure 6(c)) provide compelling evidence in favor of an early evolutionary appearance of Archaea [105, 107, 108, 130–135]. Here we briefly summarize evidence from tRNA, 5S rRNA, and RNase P RNA. Unpublished analyses of rRNA sequence and structure using advanced phylogenetic methods also show that Archaea was the first domain of life.

**Figure 8:**
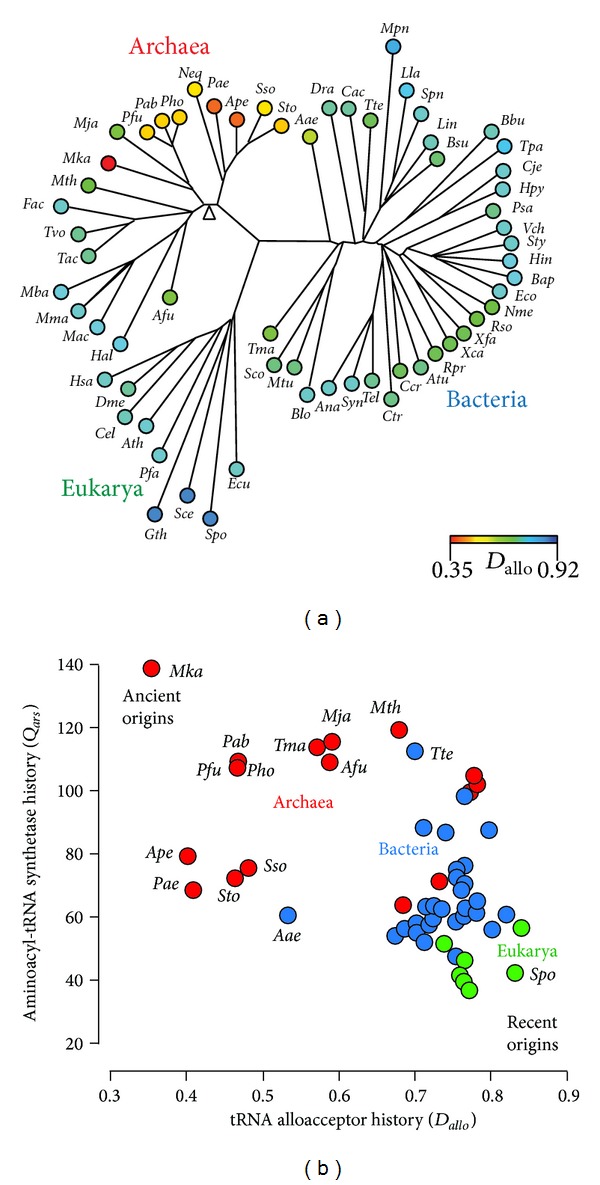
Ancient phylogenetic signal in the sequence of tRNA and their associated aminoacyl-tRNA synthetase (aaRS) enzymes. (a) Unrooted ToL derived from tRNA sequences with their alloacceptor *D*_allo_ distances traced in thermal scale (from [130]). *D*_allo_ is average of pairwise distances for 190 pairs of tRNA isoacceptors. These distances measure pairwise sequence mismatches of tRNAs for every genome and their values increase for faster evolving sequences of species of more recent origin. (b) Coevolution of aaRSs and their corresponding tRNA. Genetic distances between the top 10 potentially paralogous aaRS pairs estimated using BLASTP define a measure (*Q*_ars_) of how closely the proteins resemble each other in genomes (from [131]). Larger *Q*_ars_ scores imply more ancestral and slowly evolving protein pairs. The plot of *Q*_ars_ scores against *D*_allo_ distances reveals a hidden correlation between the evolution of tRNA and aaRSs and the early origin of Archaea (larger *Q*_ars_ and lower *D*_allo_ distances).

tRNA molecules are generally short (~73–95 nucleotides in length) and highly conserved. Consequently, their sequence generally contains limited amount of phylogenetic information. These limitations have been overcome by analyzing entire tRNomes [136], which extend the length of an organismal set of tRNAs to over 2,000 bases. Xue et al. [130, 131] analyzed the genetic distances between tRNA sequences as averages between alloacceptor tRNAs from diverse groups of tRNomes using multiple molecular base substitution models. The distances were mapped onto an unrooted phylogeny of tRNA molecules (Figure 8(a)). The “arrow of time” assumption in these studies is that ancient tRNA paralogs closely resemble each other when lineages originate close to the time of the gene duplication. Remarkably, the results revealed a paraphyletic rooting of the ToL in Archaea. The root was specifically located close to the hyperthermophilic methanogen* Methanopyrus kandleri* (Figure 8(a)). The hypothesis of this specific rooting scenario has been supported by several other studies [134, 137], including a study of genetic distances between paralogous pairs of aminoacyl-tRNA synthetase (aaRS) proteins [131]. A remarkable match between distance scores of tRNA and pairs of aaRS paralogs (Figure 8(b)) not only confirms the early appearance of Archaea but also suggests a coevolutionary trace associated with molecular interactions that are responsible for the genetic code. In fact, a recent exhaustive phylogenomic analysis of tRNA and aaRS coevolution explicitly reveals the origins and evolution of the genetic code and the underlying molecular basis of genetics [59]. Di Giulio [132, 133] has also proposed an archaeal rooting of the tree of life, specifically in the lineage leading to the phylum of Nanoarchaeota. This rooting is based on unique and ancestral genomic traits of* Nanoarchaeum equitans*, split genes separately codifying for the 5′ and 3′ halves of tRNA and the absence of operons, which are considered molecular fossils [132]. However, this claim needs additional support as contrasting evidence now recognizes* N. equitans* as a highly derived archaeal species [138].

**Figure 9:**
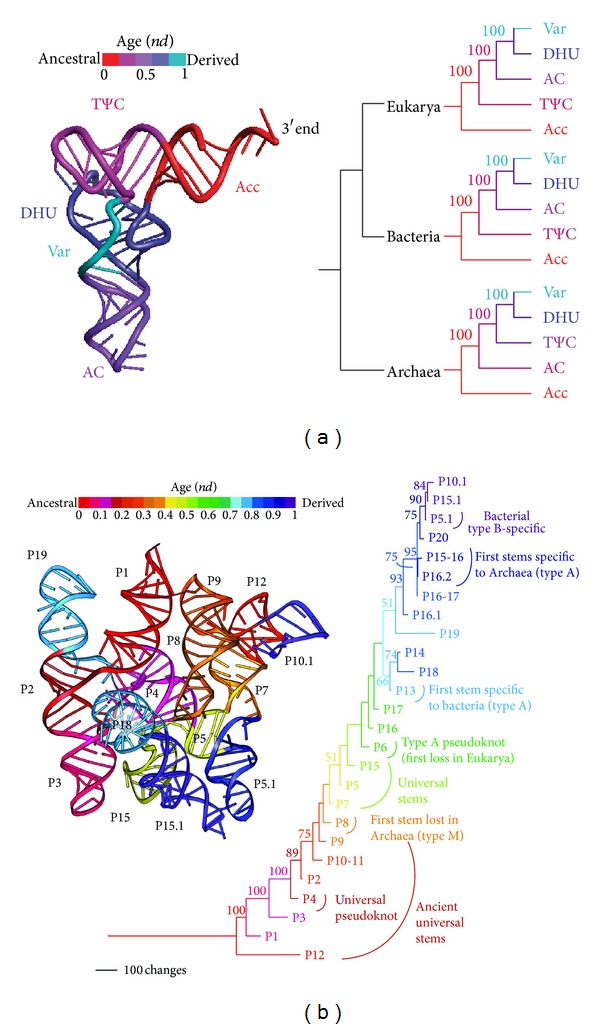
The history of accretion of tRNA and RNase P RNA substructures reveals the early evolutionary appearance of Archaea. (a) Rooted trees of tRNA arm substructures reveal the early appearance of the acceptor arm (Acc) followed by the anticodon arm (AC) in Archaea or the pseudouridine (TΨC) arm in both Bacteria and Eukarya (from [108]). The result confirms the sister group relationship of Bacteria and Eukarya. (b) Trees of molecular substructures of RNase P RNAs were reconstructed from characters describing the geometry of their structures (from [135]). Branches and corresponding substructures in a 3D atomic model are colored according to the age of each substructure (*nd*, node distance). Note the early loss of stem P8 in Archaea immediately after the evolutionary assembly of the universal functional core of the molecule.

In addition to sequences, structural features of tRNA molecules also support the archaeal rooting of the ToL. The application of RNA structural evidence in phylogenetic studies [50, 139–141] has multiple advantages over sequence data when studying ancient events, especially because RNA structures are far more conserved than sequences. This has been demonstrated in a phylogenetic approach that uses RNA structural information to reconstruct evolutionary history of macromolecules such as rRNA [29, 50], tRNA [107, 108], 5S rRNA [105], RNase P RNA [135], and SINE RNA [142]. Geometrical and statistical properties of structure (e.g., stems or loops commonly found in the secondary structures of RNA molecules) are treated as linearly ordered multistate phylogenetic characters. In order to build rooted trees, an evolutionary tendency toward conformational order is used to polarize change in character state transformations. This defines a hypothetical ancestor with which to root the ingroup using the Lundberg method. Reconstructed phylogenies produce trees of molecules and ToLs (e.g., Figure 4(a)) or trees of substructures that describe the gradual evolutionary accretion of structural components into molecules (Figure 9). For example, phylogenetic trees of tRNA substructures define explicit models of molecular history and show that tRNA originated in the acceptor stem of the molecule [108]. Remarkably, trees reconstructed from tRNA drawn from individual domains of life demonstrate that the sequence of accretion events occurred differently in Archaea than in Bacteria and Eukarya, suggesting a sister group evolutionary relationship between the bacterial and eukaryotic domains (Figure 9(a)). A similar result obtained from trees of 5S rRNA substructures revealed different molecular accretion sequences of archaeal molecules when these were compared to bacterial and eukaryal counterparts [105], confirming again in a completely different molecular system the history of the domains of life.

An analysis of the structure of RNase P RNA also provides similar conclusions [135]. While a ToL reconstructed from molecular structure placed type A archaeal molecules at its base (a topology that resembles the ToL of 5S rRNA), a tree of RNase P RNA substructures uncovered the history of molecular accretion of the RNA component of the ancient endonuclease and revealed a remarkable reductive evolutionary trend (Figure 9(b)). Molecules originated in stem P12 and were immediately accessorized with the catalytic P1–P4 catalytic pseudoknotted core structure that interacts with RNase P proteins of the endonuclease complex and ancient segments of tRNA. Soon after this important accretion stage, the evolving molecule loses its first stem in Archaea (stem P8), several accretion steps earlier than the first loss of a stem in Eukarya or the first appearance of a Bacteria-specific stem. These phylogenetic statements provide additional strong support to the early origin of the archaeal superkingdom prior to the divergence of the shared common ancestor of Bacteria and Eukarya. As we will discuss below, the early loss of a structure in the molecular accretion process of a central and ancient RNA family is significant. It suggests that the emerging archaeal lineages were subjected to strong reductive evolutionary pressures during the early evolution of a very ancient RNA molecule.

### 6.3. Phylogenomic Evidence from Protein Domain Structure

**Figure 10:**
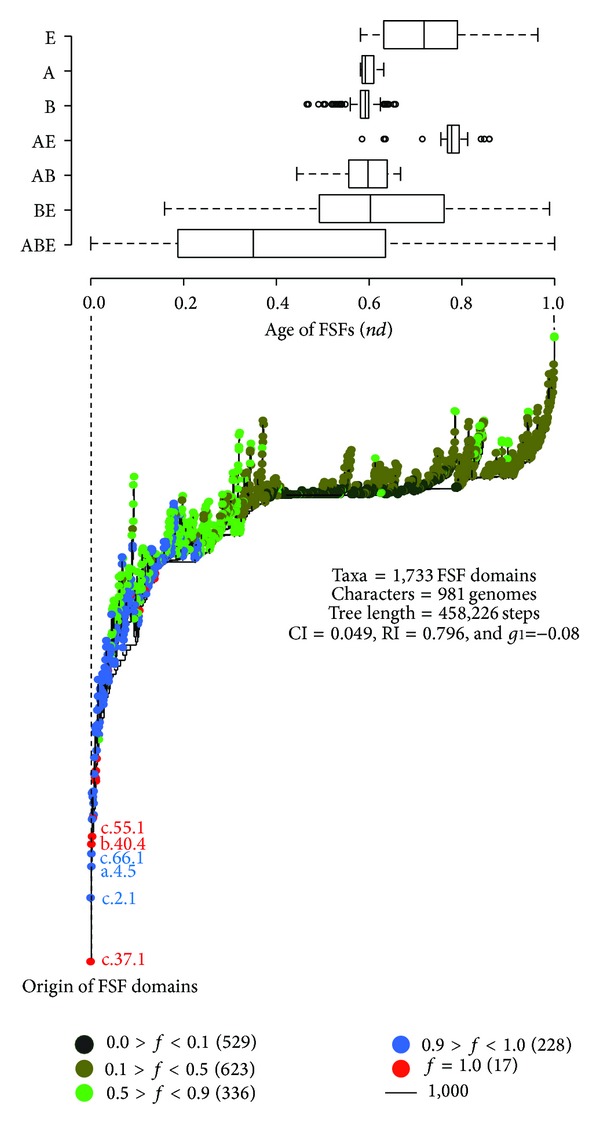
Phylogenomic tree of domains (ToD) describing the evolution of 1,733 FSF domain structures. Taxa (FSFs) were colored according to their distribution (*f*) in the 981 genomes that were surveyed and used as characters to reconstruct the phylogenomic tree (data taken from [122, 123]). The most basal FSFs are labeled with SCOP alphanumeric identifiers (e.g., c.37.1 is the P-loop containing nucleoside triphosphate hydrolase FSF). Boxplots display the age (*nd* value) distribution of FSFs for the seven possible taxonomic groups.* nd* values were calculated directly from the tree [122] and define a timeline of FSF innovation, from the origin of proteins (*nd* = 0) to the present (*nd* = 1). The group of FSFs that are shared by the three domains of life (ABE) is the most ancient taxonomic group, which spans the entire time axis and their FSFs are widely distributed in genomes. The appearance of the BE group coincides with the first reductive loss of an FSF in Archaea. FSF structures specific to domains of life appear much later in evolution.

G. Caetano-Anollés and D.Caetano-Anollés [41] were the first to utilize protein domain structures as taxa and to reconstruct trees of domains (ToDs) describing their evolution.  Figure 10 shows a rooted ToD built from a census of FSF structures in 981 genomes (data taken from [122, 123]). These trees are unique in that their terminal leaves represent a finite set of component parts [87]. These parts describe at global level the structural diversity of the protein world. When building trees of FSFs, the age of each FSF domain structure can be calculated from the ToDs by simply calculating a distance in nodes between the root of the tree and its corresponding terminal leaf. This is possible because ToDs exhibit highly unbalanced (pectinate) topologies that are the result of semipunctuated processes of domain appearance and accumulation. This node distance (*nd*), rescaled from 0 to 1, provides a relative timescale to study the order of FSF appearance in evolutionary history [126, 143]. Wang et al. [144] showed that* nd* correlates linearly with geological time and defines a global molecular clock of protein folds. Thus,* nd* can be used as a reliable proxy for time. Plotting the age of FSFs in each of the seven taxonomic groups confirmed evolutionary statements we had previously deduced from the Venn diagrams of Figure 6. The ABE taxonomic group included the majority of ancient and widely distributed FSFs. This is expected. In the presence of a strong vertical trace, molecular diversity must delimit a nested taxonomic hierarchy. The ABE group was followed by the evolutionary appearance of the BE group, which preceded the first domain-specific structures, which were Bacteria-specific (B). Remarkably, Archaea-specific (A) and Eukarya-specific (E) structures appeared concurrently and relatively late. These general trends, captured in the box plots of Figure 10, have been recovered repeatedly when studying domain structures at various levels of structural complexity, from folds to fold families [91, 126], when using CATH or SCOP structural definitions [122, 145] or when exploring the evolution of terminal GO terms.

While the early rise of BE FSFs supports the early divergence of Archaea from the urancestor, the very significant trend of gradual loss of structures occurring in the lineages of the archaeal domain and the very late appearance of Archaea-specific structures (e.g., [126]) demand explanation. Since ancient BE FSFs are widely distributed in proteomes (Figure 10), they cannot arise from separate gains of FSFs in Bacteria and Eukarya or by processes of horizontal spread of structures. This was already evident from the Venn diagrams of Figure 6. Moreover, the BE sisterhood to the exclusion of Archaea was further supported by the inspection of FSFs involved in lipid synthesis and transport (Table 1). Membrane lipids are very relevant to the origins of diversified life ([146] and references therein). Bacteria and Eukarya encode similar lipid membranes while archaeal membranes have different lipid composition (isoprenoid ethers). To check if lipid synthesis was another BE synapomorphy, we identified 17 FSFs that were involved in lipid metabolism and transport (Table 1). Remarkably, the majority of these FSFs (7 out of 17) were unique to the BE group. In comparison, none were present in either AE or AB groups. The ABE group included five universal FSFs, while one was unique to Bacteria and four were eukarya-specific. In turn, no FSF was unique to Archaea. The BE FSFs cannot be explained by modern effects impinging on variations in proteomic accumulation in FSFs or by processes of domain rearrangement, since these appear in the protein world quite late in evolution [92]. The only and most-parsimonious explanation of the patterns of FSF distribution that unfold in the ToD is the very early (and protracted) rise of the archaeal domain by processes of reductive evolution, possibly triggered by the adaptation of urancestral lineages to harsh environments and survival modes. Under extremophilic conditions typical of hyperthermophilic environments, considerable investments of matter-energy and information must be made for protein persistence [120]. This puts limits on viable protein structures [147]. Extremophilic environments will thus poise the maintenance of a limited set of FSFs for persistence of emergent diversified lineages. This would induce a primordial episode of reductive evolution in the growing FSF repertoire, explaining why hyperthermophilic and thermophilic archaeal species hold the most reduced proteomes (Figure 7). It would also explain the biases that exist in FSFs, GO terms and RNA families (Figure 6), and the placement of hyperthermophilic and thermophilic archaeal species at the base of ToLs. Since Archaea populate the oceans and sometimes rival in number Bacteria in those environments, we further interpret the late appearance of Archaea-specific FSFs as the result of late colonization of these mild environments by both ancient archaeons and emerging eukaryotes. This relaxes primordial extremophilic pressures on protein structures and enables the late archaeal exploration of structural flexibility and functional novelty.

### 6.4. Full Circle: Evidence from Trees of Proteomes and Functionomes and a Tree Derived from the Distribution of Viral Replicons in Superkingdoms

**Figure 11:**
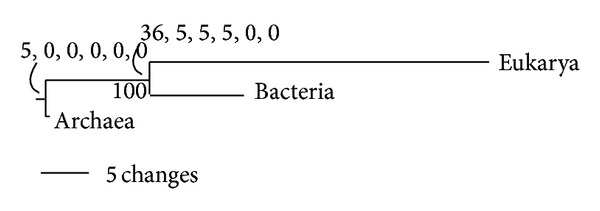
One of three optimal phylogenetic tree reconstructions with identical topologies recovered from an exhaustive maximum parsimony search (58 steps; CI = 1; RI = 1; HI = 0; RC = 1; *g*_1_ = −0.707) of the abundance of viral replicon types of dsDNA, ssDNA, dsRNA, ssRNA(+), ssRNA(−), and retrotranscribing viruses. Abundance was scored on a 0–20 scale and ranged from 0 to 759 viral replicons. Vectors of abundance reconstructions in internal nodes are given as percentage of total abundance of replicons in superkingdoms. Boostrap support values are shown below nodes.

While we distrust trees of systems, especially ToLs built from sequences, the use of molecular structure at high levels of structural abstraction has the potential to mitigate some limitations of sequence analysis [93]. For example, rooted ToLs built from abundance counts of domain structures and terminal GO terms in the genomes of free-living organisms describe the evolution of proteomes (e.g., [91]). All ToL reconstructions of these kinds approximate the physiology of living organisms, dissect the three primary domains of life, and reveal the early paraphyletic origin of extremophilic archaeal lineages, followed by the late appearances of monophyletic Bacteria and Eukarya. These patterns have been reliably recovered with datasets of varying sizes irrespective of the structural classification scheme [91, 122, 126, 145]. Even a tree reconstructed from the distribution of 2,662 viral replicons in superkingdoms from an exhaustive comparative genomic analysis of viral genomes showed the basal placement of Archaea and the sister taxa relationship between Bacteria and Eukarya (Figure 11).

### 6.5. Additional Evidence from Comparative Genomics

**Figure 12:**
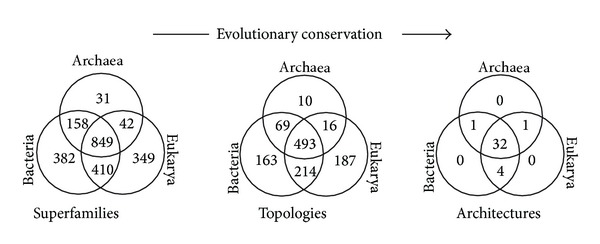
Venn diagrams displaying the distributions of 2,221 homologous superfamilies, 1,152 topologies, and 38 architectures of CATH domains in the proteomes of 492 fully sequenced genomes (from [145]). All distributions highlight maximum sharing in the ancient ABE and BE taxonomic groups and minimal sharing in archaeal taxonomic groups.

The uneven distribution of protein domain structures in the world of proteomes (Figure 6) is preserved as we climb in the structural hierarchy. This was recently made evident when studying the evolution of CATH domains [145]. The Venn diagrams of Figure 12 show how domain structures in all taxonomic groups decrease in numbers with increases in evolutionary conservation. At the highest CATH architectural level, there were 32 universal architectures but no domain-specific architectures. The four architectures shared by Bacteria and Eukarya were present in at least 60% of the proteomes that were surveyed. The other two interdomain architectures were topological designs of considerable complexity that were poorly shared between proteomes and were evolutionarily derived. They were the* clam* architectures lost in Eukarya and the* box* architectures of nucleotide excision repair shared by Archaea and Eukarya. The most parsimonious corollary of these distribution patterns is that the BE taxonomical group must arise by loss of structures in Archaea. Indeed, ToDs describing the evolution of CATH domain structures again confirm the early appearance of BE structures and consequently their loss in Archaea.

## 7. Paraphyletic Origins: Grades and Clades in Archaeal History

Saying that “a ToL is rooted in a domain of life” is an incorrect statement that comes from phylogenetic methodology (the use of outgroups) and the tendency to look at the past with modern eyes. Clades in ToLs have been rooted relative to each other by generating unrooted trees and by defining extant organisms as outgroup taxa. The ToL however must be considered rooted in the urancestor of cellular life (Figure 1). This planted edge that connects to the ingroup of extant organisms represents a cellular state in which productive diversification (in the sense of successful lineages) was absent. The primordial urancestral edge leads to a “phase transition,” the last universal cellular ancestor (LUCA), of which little is known. The physiology of the urancestor cannot be considered linked to that of any extant organism, even if it shared a common molecular core with all of them. The urancestor was not an archeon, a bacterium, or a eukaryote [148]. It was not necessarily thermophilic. Perhaps it was a communal entity or a megametaorganism in the sense of a modern syncytium (the result of multiple cell fusions) and a modern coenocyte (the result of multiple cell divisions). The organismal boundaries were likely present, judging by the number of widely distributed protein domains that associate with membranes and appear at the base of our ToDs and by the universal existence of acidocalcisome organelles [149]. However, the molecular makeup of the urancestral cells was most likely fluid and quasistatistical; the repertoire distributed unequally in the urancestral populations of communal parts, of course, within confines delimited by persistence. This urancestral population is therefore consistent with the idea of a primordial stem line proposed by Kandler and Woese [150, 151]. However, it was relatively richer in molecular structures and functions as opposed to the simple cellular systems hypothesized by Woese. This richness is confirmed by modern analyses of proteomes and functionomes that reveal vast number of universally shared protein domains and GOs among three superkingdoms. While each syncytial/coenocyte element of the megaorganism exchanged component parts in search of cellular stability and persistence, the process could not be equated with modern HGT. The exchanging community of primordial cells was not cohesive enough to make the horizontal exchange meaningful. Macromolecules most likely established loose and diverse associations with each other and with smaller molecules, limited by the short average life of their unevolved structural conformations. With time, molecules with better-optimized properties engaged in more durable interactions, stabilizing the emergent cells and providing increased cellular cohesiveness. This poised the urancestral community towards a phase transition (a crystallization; [148]), a point in which cellular groups had distinct properties and could be individuated. We believe this was the time of the origin of the archaeal lineage 2.9 billion years ago [48].

**Figure 13:**
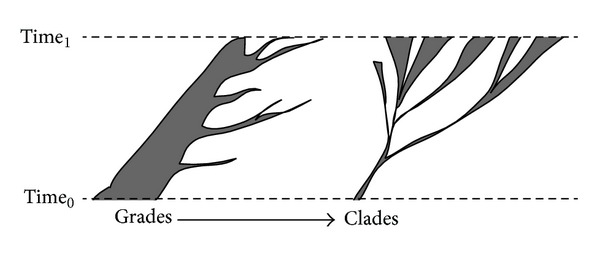
From grades to clades. The cartoon describes a possible progression of modes of organismal diversification during the rise of primordial archaeal lineages. The width of emerging lineages is proportional to uniquely identifying features of physiological and molecular complexity.

At the base of the ToLs that were reconstructed from genomic data, basal archaeal taxa arise as paraphyletic lineages (Figures 4(a) and 4(c)). These lineages likely arose from subgroups of the urancestral population that pervasively lost crucial domain structures and molecular functions. This represents an evolutionary grade under the scenario described above. The emerging lineages shared with the urancestral community a unifying condition that was related to archaic biochemistry. In other words, the urancestral and emerging archaeal lineages expressed fundamental structural and functional equivalences in terms of their repertoires, but revealed in each emerging and durable paraphyletic lineage a handful of distinct newly developed traits. These traits could be global, such as increased thermostability of some crucial members of the protein repertoire or change in the membrane makeup, or local, such as the selective loss of crucial structures and functions.  Figure 13 uses the tree paradigm to portray the structural and functional equivalences of the urancestral and emerging archaeal lineages and the slow progression from grades to clades.

## 8. Through the Wormhole: The Makeup of the “Megaorganism” and the Emerging Archaeal Lineages

Character state reconstructions of proteome repertoires derived from ToLs coupled to the timelines of ToDs provide an effective way to define the ancestral protein domain complement of the urancestor [48] and, consequently, the likely makeup of the emerging archaeal lineages. The urancestral proteome possessed a lower bound of ~70 FSF domain structures, 75% of which were composed of *α*/*β* and *α* + *β* proteins. About 50% of FSFs were part of metabolic enzymes, including a rich toolkit of transferases and enzymes of nucleotide metabolism. The rest of domains were involved in functions related to information (translation, replication, and repair), intracellular processes (transport, protein modification, and proteolytic activities), regulation (kinases/phosphatases and DNA binding functions), and small molecule binding. The urancestor had a limited repertoire of aaRSs and translation factors. It contained a primordial ribosome with a limited core of universal ribosomal proteins. It had numerous membrane proteins necessary for transport, including a relatively advanced ATP synthetase complex, and structures necessary for cellular organization (filaments and primordial cytoskeletal structures). The cells lacked enzymes for deoxyribonucleotide production, so it is likely that the cellular urancestor itself did not harbor a DNA genome. The cells lacked functions related to extracellular processes (cell adhesion, immune response, and toxins/defense) and cellular motility, suggesting an ancient living world without competitive strategies of survival.

## 9. Conclusions

The rooting of the ToL has been always controversial in evolutionary biology [26, 152, 153]. While it is popularly accepted that the ToL based on sequence phylogenies is rooted in the akaryotes and that Archaea and Eukarya are sister groups to each other, only two of the three main steps of phylogenetic analysis [104] have been partially fulfilled with sequences. This includes selecting an appropriate statistical or nonstatistical evolutionary model of character change and an optimization method for phylogenetic tree reconstruction. However, no adequate method exists for character polarization that identifies ancestral and derived character states in sequences. In the absence of robust polarization methodology, any statement about the rooting of the ToL should be considered suspect or subject of apriorism. Here we show that information derived from a genomic structural and functional census of millions of encoded proteins and RNAs coupled with process models that comply with Weston's generality criterion provide the means to dissect the origins of diversified life. The generality criterion is fulfilled in these studies by focusing on the accumulation of modules such as protein domain structures, elements of RNA substructures, or ontogenetic definitions of molecular function. In general, these features are the subject of accretion processes that comply with additive phylogenetic change within the nested taxonomic hierarchy and result in changes of abundance. These processes include those responsible for the growth of molecules (e.g., multidomain proteins), molecular ensembles (e.g., the ribosome), and molecular repertoires (e.g., proteomes). The new methods unfold a consistent evolutionary scenario in which the origin of diversified life traces back to the early history of Archaea. Remarkably, the archaic origin of this microbial urkingdom now does justice to its name.

## Figures and Tables

**Table 1:** List of FSFs involved in lipid metabolism and transport along with taxonomic distribution (data taken from [122, 123]).

Group	SCOP Id	FSF Id	FSF description
ABE	89392	b.125.1	Prokaryotic lipoproteins and lipoprotein localization factors
ABE	53092	c.55.2	Creatinase/prolidase N-terminal domain
ABE	49723	b.12.1	Lipase/lipooxygenase domain (PLAT/LH2 domain)
ABE	54637	d.38.1	Thioesterase/thiol ester dehydrase-isomerase
ABE	63825	b.68.5	YWTD domain
BE	47027	a.11.1	Acyl-CoA binding protein
BE	48431	a.118.4	Lipovitellin-phosvitin complex, superhelical domain
BE	55048	d.58.23	Probable ACP-binding domain of malonyl-CoA ACP transacylase
BE	56968	f.7.1	Lipovitellin-phosvitin complex; beta-sheet shell regions
BE	58113	h.5.1	Apolipoprotein A-I
BE	47162	a.24.1	Apolipoprotein
BE	56931	f.4.2	Outer membrane phospholipase A (OMPLA)
B	82220	b.120.1	Tp47 lipoprotein, N-terminal domain
E	47699	a.52.1	Bifunctional inhibitor/lipid-transfer protein/seed storage 2S albumin
E	82936	h.6.1	Apolipoprotein A-II
E	57190	g.3.10	Colipase-like
E	49594	b.7.4	Rab geranylgeranyltransferase alpha-subunit, insert domain
